# Uptake of a patient‐centred dynamic choice model for HIV prevention in rural Kenya and Uganda: SEARCH SAPPHIRE study

**DOI:** 10.1002/jia2.26121

**Published:** 2023-07-06

**Authors:** Jane Kabami, Elijah Kakande, Gabriel Chamie, Laura B. Balzer, Maya L. Petersen, Carol S. Camlin, Marilyn Nyabuti, Catherine A. Koss, Elizabeth A. Bukusi, Moses R. Kamya, Diane V. Havlir, James Ayieko

**Affiliations:** ^1^ Infectious Diseases Research Collaboration Kampala Uganda; ^2^ Department of Medicine Makerere University Kampala Uganda; ^3^ Department of Medicine University of California San Francisco California USA; ^4^ Department of Biostatistics University of California Berkeley California USA; ^5^ Department of Obstetrics, Gynecology & Reproductive Sciences University of California San Francisco California USA; ^6^ Center for Microbiology Research Kenya Medical Research Institute Nairobi Kenya

**Keywords:** antenatal, HIV prevention, outpatient and community, PEP, PrEP

## Abstract

**Introduction:**

Person‐centred HIV prevention delivery models that offer structured choices in product, testing and visit location may increase coverage. However, data are lacking on the actual uptake of choices among persons at risk of HIV in southern Africa. In an ongoing randomized study (SEARCH; NCT04810650) in rural East Africa, we evaluated the uptake of choices made when offered in a person‐centred, dynamic choice model for HIV prevention.

**Methods:**

Using the PRECEDE framework, we developed a persont‐centred, Dynamic Choice HIV Prevention (DCP) intervention for persons at risk of HIV in three settings in rural Kenya and Uganda: antenatal clinic (ANC), outpatient department (OPD) and in the community. Components include: provider training on product choice (predisposing); flexibility and responsiveness to client desires and choices (pre‐exposure prophylaxis [PrEP]/post‐exposure prophylaxis [PEP], clinic vs. off‐site visits and self‐ or clinician‐based HIV testing) (enabling); and client and staff feedback (reinforcing). All clients received a structured assessment of barriers with personalized plans to address them, mobile phone access to clinicians (24 hours/7 days/week) and integrated reproductive health services. In this interim analysis, we describe the uptake of choices of product, location and testing during the first 24 weeks of follow‐up (April 2021−March 2022).

**Results:**

A total of 612 (203 ANC, 197 OPD and 212 community) participants were randomized to the person‐centred DCP intervention. We delivered the DCP intervention in all three settings with diverse populations: ANC: 39% pregnant; median age: 24 years; OPD: 39% male, median age 27 years; and community: 42% male, median age: 29 years. Baseline choice of PrEP was highest in ANC (98%) vs. OPD (84%) and community (40%); whereas the proportion of adults selecting PEP was higher in the community (46%) vs. OPD (8%) and ANC (1%). Personal preference for off‐site visits increased over time (65% at week 24 vs. 35% at baseline). Interest in alternative HIV testing modalities grew over time (38% baseline self‐testing vs. 58% at week 24).

**Conclusions:**

A person‐centred model incorporating structured choice in biomedical prevention and care delivery options in settings with demographically diverse groups, in rural Kenya and Uganda, was responsive to varying personal preferences over time in HIV prevention programmes.

## 1 INTRODUCTION

Despite a significant reduction in the number of new HIV acquisitions globally, progress has slowed significantly with a drop of only 3.6% in 2021 compared to 2020 [1]. The coverage of HIV prevention interventions is still suboptimal among persons at risk of HIV highlighting the need of innovative approaches to increase HIV prevention coverage. Multiple biomedical HIV prevention options are now available, including oral pre‐exposure prophylaxis (PrEP) and post‐exposure prophylaxis (PEP), as well as long‐acting injectable cabotegravir (CAB‐LA) in some countries. Additional options in prevention service delivery include a choice of HIV testing modality (HIV rapid antibody test or self‐test) and the option for clinic‐based or out‐of‐facility delivery. Extensive literature documents the importance of offering choice as a cornerstone of patient‐centred care delivery in other health contexts, such as reproductive health services [2, 3, 4, 5, 6]. In HIV prevention, multiple discrete choice experiments (DCEs) have documented variation in stated prevention preferences, both between persons and settings, suggesting that a one‐size‐fits all approach to HIV prevention is unlikely to serve all patients well [7, 8, 9]. While existing literature supports the need to integrate patient choice as a core element of HIV person‐centred delivery models, very little literature to date documents the choices in prevention product and delivery modality that people who identify themselves as at‐risk for HIV actually make when presented with options [10, 11].

Further, effective integration of prevention options in HIV prevention delivery models requires understanding how to effectively embed choices within person‐centred care. To address these gaps, we developed a Dynamic Choice HIV Prevention (DCP) delivery model that offers structured choices in product, HIV test modality and location of service delivery, together with patient‐centred staffing, service provision and client support. Within one arm of the study, the intervention arm, we evaluated the uptake of a person‐centred, DCP model among persons at risk of HIV identified at antenatal clinics (ANC), outpatient departments (OPD) and in the community in rural Uganda and Kenya (SEARCH: NCT04810650).

## 2 METHODS

### 2.1 Study setting, design and population

The study population includes persons randomized to the intervention arms of three ongoing pilot trials to evaluate the effect of DCP intervention versus the standard of care. The studies are being conducted in some of the highest seroprevalence areas in rural Southwestern Uganda and Western Kenya [12, 13]. The first trial recruited participants presenting to ANC; the second trial recruited from the OPD (primary care clinics) and the third trial recruited from the community (eight in Uganda villages and another eight in Kenya).

The inclusion criteria for the ANC, OPD and community trials were the same: HIV‐negative status, age 15 years or more and current or anticipated HIV risk. Baseline HIV risk was assessed by asking potential participants if they were at risk for HIV using the country Ministry of Health PrEP screening tool and self‐assessment. The Ministry of Health screener was country‐specific and included questions about having a partner with HIV, diagnosis of a sexually transmitted infection, repeated use of PEP and sex in exchange for money (Supporting Information: SAPPHIRE risk screening tool). Additionally, we asked participants to self‐assess if they were currently at risk or anticipated being at risk in the next 3 months. Exclusion criteria were age less than 15 years, inability to provide consent or participation in another Sustainable East Africa Research in Community Health (SEARCH) study. Eligible participants were randomized to the patient‐centred DCP intervention, described next, or the control, which included standard referrals to HIV prevention services.

### 2.2 Study intervention

The person‐centred “Dynamic Choice HIV Prevention” (DCP) implementation strategy for delivering existing evidence‐based, biomedical prevention interventions was developed using the PRECEDE framework for health promotion strategies to address “predisposing” factors (i.e. knowledge, attitudes or beliefs) that impact behaviour, “enabling” factors to facilitate behaviour and “reinforcing” factors that include consequences of following a behaviour (Table 1). Intervention components were selected based on qualitative and survey data and include structured choices in biomedical prevention product, HIV test modality and location of service delivery, together with person‐centred staffing, service provision and client support. Specifically, the DCP model offers participants choices on prevention modality on an ongoing basis: oral PrEP or oral PEP, and the option to switch between products.

**Table 1:** Person‐centred, Dynamic Choice Prevention (DCP) delivery model.

Intervention	Population and frequency of delivery	Purpose
**Education, case studies and discussion** on concept of dynamic prevention and on the profile of each prevention option product	Health centre leadership and staff, clinicians, provided the initial and ongoing training and education to the study participants.	Predisposing
**Dynamic Choice Prevention (DCP) package** (risk assessment and choice of product, HIV testing, service delivery site and refill duration) integrated into ANC and OPD clinics, and through routine community health worker visits. Clients also receive support services for reproductive health and gender‐based violence, travel packs and access to a 24‐hour hotline for client logistical or medical questions.	Study participants at the visits to ANC, visits to OPD and in the communities served by the community health workers are offered the DCP with scheduled check‐ins every 3 months or more frequently based on participants choice.	Enabling
**Provider text or phone check‐in to participant** 1 week after starting new prevention product option, and supportive adherence counselling.	Participants are provided with a phone contact of the clinician/provider to consult and ask any questions during the study. This contact is available 24 hours/7 days per week. In addition, staff contact all participants who initiate PrEP or PEP by phone to assess adherence and any other concerns every 2 weeks in the first month, and monthly thereafter.	Reinforcing

Abbreviations: ANC, antenatal clinics; OPD, outpatient departments.

The intervention is being delivered using a person‐centred approach designed to be sensitive and responsive to the choice and preference of the clients. The intervention is being delivered by clinical officers and nurses in the ANC and OPD and by community health workers (CHWs) who facilitate intervention by clinical officers from the local health centre in the community trial. All clinical and community health team staff (i.e. clinical officers, nurses, coordinators and health workers) are trained and equipped for HIV prevention care in the clinical setting, appropriate to their role. Service delivery is deliberately designed to be offered in a warm and friendly atmosphere aimed at making clients feel comfortable during the participant—provider interactions. The intervention is designed to enhance flexibility and convenience by presenting choice to participants with the following components:

- Biomedical product choice: the option of oral PrEP or PEP.
- Service location choice: the options of the location of service delivery, including home, clinic, other community locations and phone/virtual visits.
- Testing choice: the options of HIV rapid blood test and oral‐based self‐testing (HIVST) with clinician‐assisted testing in cases where participants need help during self‐testing.
- Refill duration choice: the option to select the duration of their refill (1−3 months) based on their personal preference which hinges on factors, such as travel.

### 2.3 Measures

Demographics and self‐reported use of any PrEP or PEP in the prior 6 months were collected by survey at the study baseline. At intervention visits weeks 4, 12 and 24, participant selection of structured choice of prevention option (PrEP, PEP, condoms only and no selection), HIV testing modality (oral self‐test or clinician administered rapid antibody) and preferred location for next visit (clinic vs. out‐of‐facility) was recorded. At week 24, PrEP and PEP use and HIV risk (report of sexual partners with HIV or unknown status and/or self‐identification as being at risk) for each of the prior 6 calendar months were assessed via a structured survey. Enrolment began in April 2021, and the data collection for week 24 concluded in March 2022.

### 2.4 Analysis

Visit attendance was assessed at weeks 4, 12 and 24 among participants enrolled in the three trials. We excluded all participants who seroconverted and withdrew from the trial. We evaluated the proportion of participants selecting each DCP option at each scheduled visit, and the proportion of participants who ever selected PrEP and PEP during 24‐week follow‐up at each of the three settings. The proportion of follow‐up time covered by biomedical prevention (“biomedical covered time”) for a given participant was calculated as the number of months during which a participant reported PrEP or PEP use divided by the number of months for which self‐reported use was assessed. Participants who acquired HIV were assumed not to be covered during the period prior to seroconversion. “At risk” biomedical covered time was calculated analogously, but restricted to months for which a participant reported HIV risk. We report mean, median, first quartile (Q1) and third quartile (Q3) of both measures across participants.

### 2.5 Ethical considerations

Ethical approval to conduct the study was received from the University of California, San Francisco Committee on Human Research (UCSF—Sept 2020), Makerere University School of Medicine Research and Ethics Committee (SOMREC—March 2021), Uganda National Institute of Science and Technology (UNCST—April 2021) and the Scientific Ethical Review Unit of the Kenya Medical Research Institute (KEMRI—April 2021). All participants involved provided written consent to participate in the study.

## 3 RESULTS

### 3.1 Study population

A total of 612 (203 ANC, 197 OPD and 212 community) participants were randomized to the person‐centred prevention intervention (Table 2 and Figure S1). The most common job was farming (ANC 32%, OPD 39% and community 42%); a substantial minority were students (9%, 15% and 18%, respectively). ANC participants were younger (52% aged 15–24 years) than participants in the OPD and community settings (39% and 36% aged 15–24 years, respectively). In the OPD and community trials, 39% and 42% of participants were male; 38% of ANC participants were pregnant at baseline. Despite the self‐reported risk of HIV at study start, fewer than 10% of participants reported any use of PrEP or PEP in the 6 months prior to study enrolment (5% ANC, 10% OPD and 2% community).

**Table 2:** Baseline characteristics of 612 participants enrolled in the person‐centred Dynamic HIV Choice Prevention (DCP) intervention in three trials: antenatal clinic (ANC), outpatient department (OPD) and the community.

	ANC	OPD	Community	Total
	*n* = 203	*n* = 197	*n* = 212	*N* = 612
**Country**, *n* (%)				
‐Kenya	103 (51)	97 (49)	110 (52)	310 (51)
‐Uganda	100 (49)	100 (51)	102 (48)	302 (49)
**Age 15–24**, *n* (%)	106 (52)	76 (39)	76 (36)	258 (42)
**Male**, *n* (%)	0 (0)	77 (39)	88 (42)	165 (27)
Occupation^a^, *n* (%)				
‐Farmer	64 (32)	76 (39)	88 (42)	228 (37)
‐Student	18 (9)	30 (15)	37 (18)	85 (14)
‐Shopkeeper/market vendor	26 (13)	19 (10)	17 (8)	62 (10)
‐Housewife	33 (16)	4 (2)	11 (5)	48 (8)
‐No job	14 (7)	23 (12)	7 (3)	44 (7)
‐Manual labour/construction	1 (0)	7 (4)	11 (5)	19 (3)
‐Fishing/fishmonger	4 (2)	1 (1)	4 (2)	9 (1)
‐Other	42 (21)	36 (18)	36 (17)	114 (19)
**Marital status** ^a^, *n* (%)				
‐Single (unmarried)	49 (24)	51 (26)	64 (30)	164 (27)
‐Married/cohabitating	154 (76)	136 (70)	134 (63)	424 (70)
‐Divorced/separated/widowed	0 (0)	8 (4)	14 (7)	22 (4)
**Alcohol use** (any, prior 3 months), *n* (%)	14 (7)	24 (12)	20 (9)	58 (9)
**Nights away** ^a^ in past 3 months, median [Q1,Q3]	0 [0,0]	0 [0,3]	0 [0,3]	0 [0,2]
**Pregnant** ^a^ (female only), *n* (%)	80 (39)	3 (3)	11 (9)	94 (21)
**Used PrEP/PEP** in past 6 months, *n* (%)	11 (5)	19 (10)	5 (2)	35 (6)

^a^ Missing occupation for three participants, marital status for two participants, mobility (nights away) for 26 participants and pregnancy (among women) for seven participants.

Abbreviations: PrEP, pre‐exposure prophylaxis; PEP, post exposure prophylaxis.

### 3.2 Visit adherence

Between baseline and week 24, 202/203 (99.5%) of participants in ANC, 192/197 (97.5%) in OPD and 210/212 (99.1%) in community settings remained eligible for intervention delivery (four withdrew and four seroconverted; zero died). At week 4 following randomization, 84% of ANC, 89% of OPD and 98% of eligible community participants were seen and offered a dynamic choice of product, test modality and location for the next visit. Visit adherence remained high across all trial settings at weeks 12 (95% ANC, 92% OPD and 91% community participants seen) and 24 (92% ANC, 89% OPD and 89% community).

### 3.3 Selections among dynamic prevention choices over time

**Figure 1:**
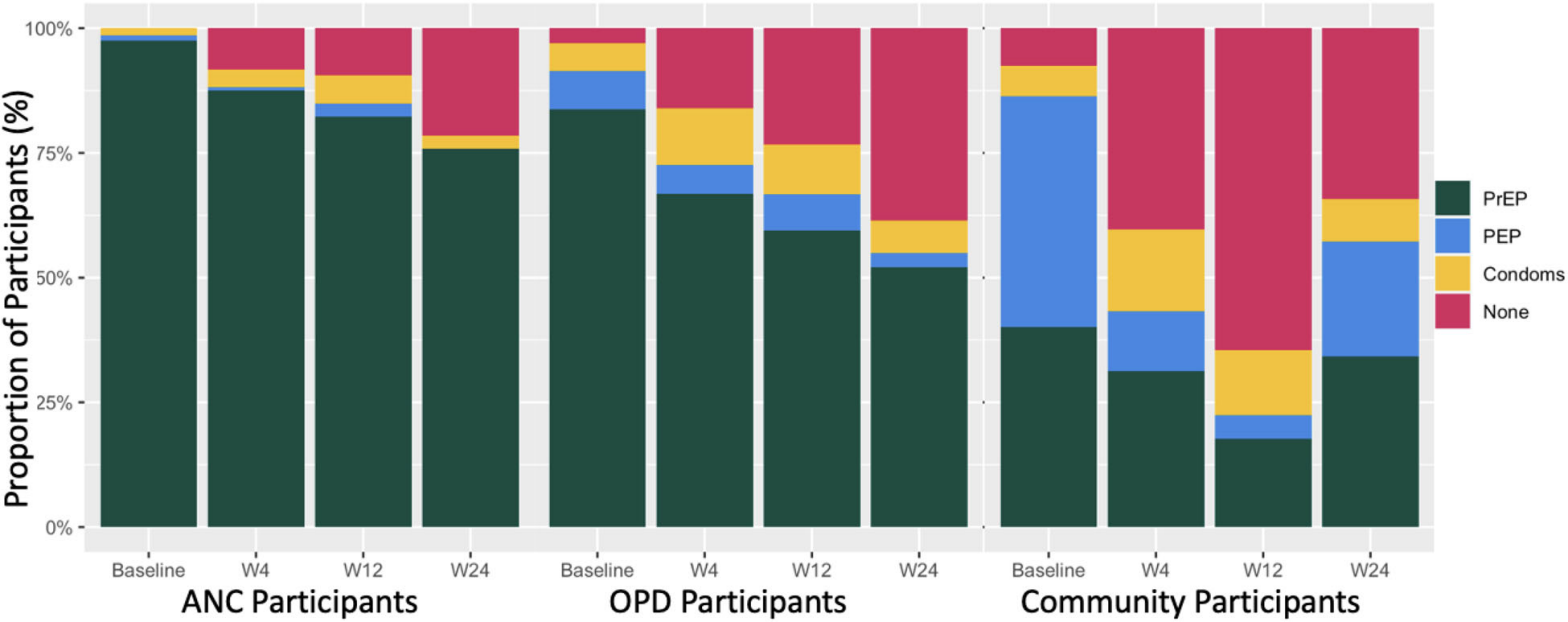
Choice of prevention options: (PrEP—pre‐exposure prophylaxis; PEP—post exposure prophylaxis, condoms or nothing) with each bar representing choices among participants seen at baseline, week 4 (W4), week 12 (W12) and week 24 (W24) in the ANC (left), OPD (middle) and community (right) settings. The different colours represent the different preferences and choice of prevention option. Abbreviations: ANC, antenatal clinics; OPD, outpatient departments.

At baseline, PrEP was selected as an initial prevention product by 98% of participants in ANC, 84% of participants in OPD and 40% of participants in the community (Figure 1); over the course of the 24‐week follow‐up, 100% of ANC, 86% of OPD and 50% of community participants selected PrEP at least once. The initial choice of PEP for HIV prevention was highest in the community setting (46%) compared to the OPD and ANC settings (9% and 1%, respectively). Selection of PEP remained highest in the community setting over time (23% at week 24); in the ANC and OPD settings, only 3% and 11%, respectively, ever selected PEP. In all settings, participant selection of an active biomedical prevention product (PrEP or PEP) declined over time (97% of ANC, 55% of OPD and 57% of community participants at week 24 selected either PrEP or PEP).

Participants from the three study settings differed in preference for visit location; off‐site delivery of prevention services was initially selected by 93% of community participants, compared to 22% of ANC and 8% of OPD participants. Personal preference for off‐site visits remained high in the community setting (99% at week 24) and increased over time in ANC and OPD (with 51% in ANC and 36% in OPD opting for off‐site delivery at week 24). Across the trials, the most common choice for off‐site visits was homes (86%), followed by phone/virtual visits (7%), trading centres (2%) and schools (2%).

At baseline, HIV self‐testing was selected by 34% of ANC participants, 26% of OPD participants and 52% of community participants. In all three settings, personal/individual interest in alternative HIV testing modalities increased over time (57% ANC, 52% OPD and 65% community at week 24).

### 3.4 Biomedical covered time and dynamic risk

**Figure 2:**
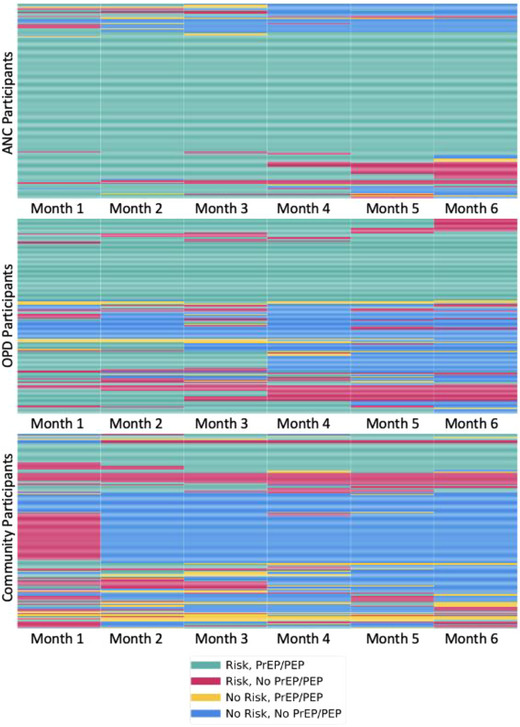
Heat maps of use of biomedical prevention by HIV risk over the 24‐week follow‐up period in the ANC (top), OPD (middle) and community (bottom) settings. Each row corresponds to a participant, and each column to a follow‐up month. Green represents HIV risk with biomedical coverage (i.e. use of oral PrEP or PEP); red represents HIV risk without biomedical coverage; yellow represents no HIV risk but with biomedical coverage; and blue represents no HIV risk and no coverage. Abbreviations: ANC, antenatal clinics; OPD, outpatient departments.

At week 24, the structured survey to assess the use of PrEP or PEP and HIV risk over the prior 6 months was completed by 91% (554/612) participants overall: 94% ANC participants, 87% OPD participants and 90% community participants. Mean biomedical covered time (proportion of 24‐week follow‐up during which a participant reported the use of either PrEP or PEP) was 80% in ANC (median 100%, Q1: 67%, Q3 100%), 60% in OPD (median 67%, Q1 33%, Q3 100%) and 32% in the community setting (median 0%, Q1 0%, Q3 67%). While all participants reported current or anticipated HIV risk at baseline, self‐reported HIV risk experienced, assessed retrospectively at week 24, varied over time (Figure 2). Across the three trials, an average of 88% of follow‐up time at risk of HIV was covered by PrEP or PEP use in the ANC trial (median 100%, Q1 100%, Q3 100%), as compared to 75% in OPD (median 100%, Q1 50%, Q3 100%) and 42% in the community setting (median 17%, Q1 0%, Q3 100%).

## 4 DISCUSSION

We implemented a person‐centred model for dynamic choice in HIV biomedical prevention in three distinct settings with demographically diverse groups and found that uptake of intervention components, including product, product delivery and HIV testing modality, varied between locations and over time. This model was responsive to client preferences and resulted in higher retention in prevention services than has been observed in previous studies conducted among subgroups of high acquisition risk [14, 15].

We observed the highest uptake of biomedical prevention among women receiving services at ANC. Reflecting ongoing HIV risk, PrEP was the preferred option for nearly all women. As has been reported by others, PrEP use waned over time [16]. A Maternal Child Health ANC clinic platform for PrEP delivery presents built‐in advantages, such as existing services for the prevention of perinatal transmission and integrated HIV testing and retesting of women living with HIV, as has been noted in previous studies [16]. These may have contributed to the high uptake and retention observed as compared to the OPD and community delivery approaches. Our ANC model further presented a choice of service location delivery and testing, women increasingly chose to receive PrEP via out‐of‐clinic delivery options over time, and utilize self‐testing which enabled them to engage in biomedical prevention and monitor for HIV without having to travel to a clinic. This option may have been particularly convenient post‐partum, when women were caring for one or more newborn infants. Previous studies have reported increasing uptake of self‐testing [17] due to convenience. In the Partners Demonstration Project sub‐study, participants reported that HIVST between PrEP clinic visits reduced anxiety while waiting to return for a PrEP clinic visit [18]. In the Empower study, participants felt that HIVST between PrEP clinic visits empowered them economically by reducing costs of visiting the clinic for testing, restored trust and intimacy between sexual partners, addressed barriers, such as stigma, associated with accessing sexual health services and encouraged behaviours that prevent HIV acquisition, such as condom use [19]. Most recently, the JiPime‐JiPrEP trial found that adherence and visit attendance were non‐inferior among persons randomized to 6‐monthly visits with HIVST versus standard of care [20].

Like in the ANC setting, persons in the OPD setting also preferred oral PrEP with a small proportion opting for PEP as the prevention option of choice at subsequent study visits. Surprisingly, unlike the ANC that has inbuilt retention mechanisms for subsequent pregnancy‐related follow‐up visits, we still observed a high proportion of participants accessing prevention at the OPD clinic setting, which may have been as a result of the patient‐centred care delivery model. Participants also increasingly opted for out‐of‐facility delivery over time, possibly allowing for retention of those who would potentially have dropped off from care if service access was restricted to the clinic. As observed in the ANC clinic, the proportion of participants using the self‐testing option increased with time, enhancing convenience and engagement in continued access to prevention services. We speculate that our uptake and retention was high as compared to other PrEP studies because we offered PrEP in HIV‐status‐neutral settings such as OPD and ANC as opposed to the standard practice of offering PrEP at the HIV clinic in these rural settings, a practice that is associated with increased stigma towards PrEP acknowledging that fear or worry of stigma have been expressed as motivations not to use PrEP [21].

In the community setting, overall uptake of biomedical prevention was much lower than in the two clinic‐based settings. Unlike studies conducted using a community mobile clinic or at community locations besides the household that reported high acceptability [22, 23], our model delivered prevention at the household. We posit that the uptake was lower in this model compared to ANC and OPD because the household setting may not present a conducive environment to explore HIV risk and the selection and uptake of appropriate interventions because of unintended discussion of risky sexual behaviour to other family members in a largely conservative rural context. We observed the highest proportion of PEP as the choice for prevention at baseline for the community model when compared to the ANC and OPD, but limited use of PEP during follow‐up.

Our dynamic choice model included options for product, testing and delivery on the background of supportive patient‐centred services. Training of providers and CHWs on offering choices without imposing their own views on what might be best for the client was an important part of the intervention. This training included not only the principles of choice but also case studies to illustrate how providers can support the agency for client decision‐making. The training emphasized the delivery of warm patient‐friendly services to foster provider−client trust in discussing HIV risk and the best available option without fear of feeling judged. All providers were trained on patient‐centred delivery prior to the baseline visit. There were monthly meetings of providers, as well as scheduled on‐job booster trainings during the course of the study.

Our dynamic choice model increased biomedical covered time during self‐reported HIV risk, but fell short of optimal coverage. The opportunity to add novel, emerging biomedical prevention products such as CAB‐LA as one of the choices for prevention holds promise to increase HIV prevention covered time with this option that has been shown to have higher efficacy than oral PrEP and an ability to confer protection over an 8‐week period following a single administration [24]. Previous studies have reported daily oral pill fatigue, forgetting to take the pill and the stigma associated with taking antiretroviral pills as some of the major barriers to uptake and adherence to oral PrEP [25, 26]. Injectable CAB‐LA surmounts these barriers and is expected to increase prevention coverage for those at risk by altering the route and frequency of PrEP administration [28]. Furthermore, it is expected to enhance convenience and broaden the range of options in the HIV prevention toolkit [26, 27]. Reassuringly, CAB‐LA trials have demonstrated safety with minor side effects being reported, the most common being injection site reactions that tended to decrease over time [24]. Presenting CAB‐LA in different settings in the context of a patient‐centred choice model holds promise to increase prevention coverage further for persons at risk of HIV exposure.

Our study has a number of strengths and weaknesses. It is among the first to provide evidence from the real world on biomedical choices selected when offered in different contexts (in contrast to theoretical choices via DCEs). Moreover, this study provides evidence of the implementation of PrEP and PEP in ANC, in OPD clinics (primary care settings) and in the community through a CHW‐led model in regions with high HIV prevalence. It presented an opportunity to explore innovative delivery approaches and demonstrate the value of choice in HIV prevention. Limitations of this study include the short duration of follow‐up and reliance on self‐report. In other words, recall bias is a potential concern, which we aimed to minimize by including prompts in our surveys and limiting them to discrete periods (i.e. months). Additionally, the ongoing trial is confirming that clients were actually ingesting PrEP and PEP with objective biomarkers. In this interim analysis, we are able to show that prevention coverage increased from baseline over 24 weeks among intervention participants, but the comparison to a contemporary control population is lacking in this analysis. Upon each trial's completion, we will compare biomedical covered time, overall and during periods of risk, by the randomized arm; this will help quantify the effect of this model on uptake and retention over a longer duration. These results combined with ongoing qualitative studies of provider and client attitudes can shed light on contributions of various elements of our intervention.

## 5 CONCLUSIONS

This is one of the first studies to systematically offer a structured intervention for biomedical prevention options using a theory‐based, person‐centred dynamic choice model that adapted services based on client risk and life circumstances over time. This interim analysis demonstrated the intervention was successfully delivered in a variety of settings that are entry points for HIV prevention and can be adapted as new prevention options such as CAB LA become available.

## COMPETING INTERESTS

The authors report no competing interests in this work.

## AUTHORS’ CONTRIBUTIONS

JK, EK, CAK, MN and JA contributed to the study design, data analysis and interpretation, literature search and writing of the manuscript. LBB and MLP contributed to the study design, data analysis and interpretation, literature search and writing of the manuscript. MRK, GC and DVH contributed to the study design, data interpretation and writing of the manuscript. EAB and CSC contributed to the interpretation of the data and writing of the manuscript. All authors have read and approved the final version.

## FUNDING

Research reported in this manuscript was supported by the U.S. National Institute of Allergy and Infectious Diseases (NIAID), the National Heart, Lung, and Blood Institute (NHLBI) and the National Institute of Mental Health (NIMH) and co‐funded under award number U01AI150510.

### DISCLAIMER

The content is solely the responsibility of the authors and does not necessarily represent the official views of the NIH.

## Supporting information


**Supporting Information**: SAPPHIRE risk screening tool

**Supporting Information Figure S1**: CONSORT Diagram

## Data Availability

The data that support the findings of this study are available on request from the corresponding author. The data are not publicly available due to privacy or ethical restrictions.
