# Supplementary material for: Uptake of a patient‐centred dynamic choice model for HIV prevention in rural Kenya and Uganda: SEARCH SAPPHIRE study
Source: J Int AIDS Soc. 2023 Jul 6;26(Suppl 1):e26121. doi: 10.1002/jia2.26121 (PMC10323314; doi:10.1002/jia2.26121)
Supplement: Supplementary file 1 — Supporting Information: SAPPHIRE risk screening tool [file JIA2-26-e26121-s002.docx]

**Supplementary Figure S1**. CONSORT Diagram

|  |
| --- |
| **Allocated to standard of care (n=620)**  **Allocated to DCP intervention (n=612)**    Assessed for eligibility (n=3073) |

Excluded (n=1841)

♦  No HIV risk reported (n=1339)

♦  With HIV (n=135)

♦  Declined participation (n=335)

♦  Otherwise (n=32)

Included in analysis (n=612)

Randomized (n=1232)
