# Supplementary material for: Uptake of a patient‐centred dynamic choice model for HIV prevention in rural Kenya and Uganda: SEARCH SAPPHIRE study
Source: J Int AIDS Soc. 2023 Jul 6;26(Suppl 1):e26121. doi: 10.1002/jia2.26121 (PMC10323314; doi:10.1002/jia2.26121)
Supplement: Supplementary file 2 — Supporting Information Figure S1: CONSORT Diagram [file JIA2-26-e26121-s001.docx]

**Supplemental information: SEARCH SAPPHIRE Study screening Tool**

For all participants who met the general inclusion criteria and willing to consent to participate in the study a risk assessment/screening was conducted using the following questions

A. For every potentially eligible participant, proceed with risk screening questions below.

- First, ask if the client has been sexually active in the past 6 months. Potential responses are yes/no
- Then, ask if the client has a partner who is HIV-positive. Potential responses are yes/no/don’t know
- Then, ask the following MoH PrEP screening tool
- questions – if the response to ANY question is “yes” skip to the next section
- Have you had condomless sexual intercourse with more than one partner of unknown HIV status in the past six months?
- Are you unable or unwilling to consistently use condoms AND member of one of the following groups?
  - - young woman ages 15-24
    - fisherfolk
    - boda boda driver
    - long-distance truck driver
    - uniformed services personnel
    - sex worker
    - MSM
- Have you had more than one episode of a STI within the last twelve months?
- Have you used post-exposure prophylaxis (PEP) more than 3 times in one year?
- Have you had sex in exchange for money, goods or a service in the last six months?
- Have you used recreational drugs, especially injectable drugs, in the last six months?
- Has had anal sexual intercourse in the last 6 months.
- Is part of a discordant couple, especially if the HIV positive partner is not on ART or has been on ART for less than 6 months or is not virally suppressed.
- Are members of a key or priority population who are unable or unwilling to achieve consistent use of condoms.

B. Regardless of the answers to the above questions, all clients were asked the next 2 questions on SEARCH risk screen:

Do you think you are currently at risk for HIV infection?

Do you think you will be at risk for HIV infection in the next 3 months?

*Study inclusion criteria: Must meet all criteria for general participation*

*AND*

- *At least 1 question in Section A OR Section C = YES*
- If a potentially eligible participant meeting the HIV status inclusion criterion has none of the above risks for HIV, tick ‘No’ at the bottom of the form to indicate inclusion criteria was not met, and thank the participant for their time.
- If the participant is to be included in the trial, The provider will then fill the following subject consent section by ticking the appropriate response on the section: N/A if subject is ineligible or declined prior to consent discussion. They will also assess that the consenting staff completed the following tasks
